# Conducting a diabetes mellitus prevention trial in women with GDM in Pakistan: a feasibility study

**DOI:** 10.1186/s40814-024-01514-3

**Published:** 2024-06-15

**Authors:** Romaina Iqbal, Sabahat Naz, Sana Sheikh, Rahat Qureshi, Shereen Bhutta, Haleema Yasmeen, Iqbal Azam, Paramjit Gill

**Affiliations:** 1 Community Health Sciences Department, The Aga Khan University, Karachi, Pakistan; 2 Department of Medicine, The Aga Khan University, Karachi, Pakistan; 3 Department of Obstetrics and Gynecology, The Aga Khan University, Karachi, Pakistan; 4 Jinnah Postgraduate Medical Center, Karachi, Pakistan; 5 Division of Health Sciences, University of Warwick, Coventry, UK

**Keywords:** Gestational diabetes mellitus, Lifestyle modification, Primary prevention, Pakistan

## Abstract

**Background:**

Women with gestational diabetes mellitus (GDM) are at a greater risk of developing type 2 diabetes mellitus (T2DM) than women without GDM. Despite this elevated risk, few trials on the prevention of T2DM among South Asian women with GDM have been reported. Therefore, this study aimed to assess the feasibility of conducting a diabetes prevention program on women with a history of GDM to inform the development of a contextually relevant definitive trial.

**Methods:**

Using a randomized controlled trial, women with GDM (*n* = 180) who delivered at the study hospitals (one public and one private teaching hospital, Karachi) with fasting blood glucose levels < 120 mg/dl at 6 weeks postpartum were randomized to the intervention (*n* = 88) or control arms (*n* = 92). Women in the intervention group received individualized home-based educational sessions from trained community health workers at 0, 1, 3, 6, and 9 months. In addition, they received short text messages, prerecorded messages, and printed educational material (calendars and pamphlets) for reinforcement. The intervention was centered on equipping women with knowledge, skills, and confidence to eat a healthy diet rich in fruits, vegetables, and low-fat dairy products and perform regular physical activity based on walking and household chores to reduce weight (up to 5% of their initial body weight). Women in the control arm received standard care. The feasibility outcomes of the study included screening, recruitment, and retention rates and in-depth interviews at 6 months post-intervention to explore women’s experiences with the intervention. Descriptive analysis and thematic analysis were performed.

**Results:**

Of the 324 women screened during the antenatal care visits and after delivery, 255 (78.7%) were contactable 6 weeks postpartum, and 180 (70.6%) were eligible and randomized to intervention (*n* = 88) and control (*n* = 92) groups. Loss to follow-up in the intervention and control arms was 22.7% (*n* = 20/88) and 18.5% (*n* = 17/92), respectively. Women expressed satisfaction with home-based counseling and follow-up visits, text message reminders, and printed material in the form of a calendar through our qualitative interviews.

**Conclusions:**

Home-based lifestyle modification intervention augmented with text messages and printed material is feasible. However, to evaluate the intervention’s effectiveness, a larger trial is warranted to assess its long-term impact on diabetes prevention.

**Trial registration:**

ISRCTN, ISRCTN11387113. Registered 5 December 2017—retrospectively registered.

**Supplementary Information:**

The online version contains supplementary material available at 10.1186/s40814-024-01514-3.

## Key messages regarding the feasibility

- Despite the increased risk of T2DM among women with GDM, there is a notable scarcity of trials focusing on preventing T2DM among South Asian women with GDM.
- The proposed lifestyle modification intervention was found feasible among women with recent GDM with consistent guidance on diet and physical activity, coupled with home visits and calendars, which were notable strengths of the intervention.
- The feasibility trial supported the expansion of this work with some modifications to equip high-risk women with the essential knowledge, skills, and guidance to make well-informed decisions regarding their health and overall well-being.

## Background

Diabetes continues to be a rising global public health concern, with 537 million people between 20 and 79 years of age living with diabetes worldwide [1]. The number is projected to increase by 643 million and 783 million by 2030 and 2045, respectively [1]. In addition, approximately 10–25% of pregnancies experience hyperglycemia globally [2], which makes an additional 21 million cases to the global diabetes burden [3]. Gestational diabetes mellitus (GDM) refers to any form of glucose intolerance that is recognized for the first time during pregnancy or subsequent pregnancies [4]. GDM risk factors include a family history of diabetes, age > 30 years, overweight/obese at the time of conception, and South Asian origin [5]. Pregnancies complicated by GDM have increased incidences of fetal [3] and childhood obesity [6] with associated long-term risks [7]. The effects of GDM are life-long, with women having a > 70% lifetime risk of developing type 2 diabetes mellitus (T2DM) [7].

In low- and middle-income countries (LMICs), such as Pakistan, three in every four people living are affected by diabetes [1]. Pakistan is also going through a nutrition transition and grappling with the double burden of disease [8]. The prevalence of diabetes in Pakistan is 16.9% and is significantly higher in females than males (17.8% vs. 16.2%) [9]. Pakistan’s global ranking for people with diabetes is currently 4th and is expected to reach 3rd place by 2045 [3]. The prevalence of GDM in Pakistan is 11.8% [10], with 14.1% of women progressing to T2DM 2 years after GDM [11]. Therefore, there is an urgent need to implement a coordinated approach to prevent T2DM among women with a history of GDM. It has been established that weight loss-targeted lifestyle modification interventions, including a healthy diet and moderate physical activity, can reduce T2DM risk by up to 58% in high-risk people [12]. Therefore, we conducted a feasibility study in Karachi, Pakistan, on women with a history of GDM to prevent the development of T2DM and inform the development of a contextually relevant definitive randomized controlled trial (RCT).

## Study aims

- To assess the feasibility of the trial through screening, recruitment, and retention rates in a diabetes prevention program among women with a history of GDM in Karachi, Pakistan
- To explore women’s experience with the intervention in a diabetes prevention program (process evaluation)
- To estimate the change in the anthropometric (weight and body fat %), behavioral (diet), and metabolic outcomes (blood pressure, blood glucose levels, and cholesterol levels), which may inform a sample size calculation for a future definitive randomized controlled trial

## Methods

### Study design, setting, and duration

The feasibility study was a mixed-methods, open-label, two-arms, randomized controlled trial conducted in one private and one public hospital in Karachi, Pakistan. The enrollment commenced in January 2016, and women were followed for one year till December 2017. Both hospitals were tertiary-level teaching hospitals catering to a large proportion of the city's population. They account for 15,000 deliveries per year in Karachi. First, a feasibility trial with individual-level randomization was conducted on potential participants, followed by in-depth interviews (IDIs) to explore women’s experiences with the intervention.

### Screening and recruitment of participants

We performed screening at two stages. In the first stage of screening, women admitted to the obstetrics wards after delivery were approached in the private hospital. In contrast, women who attended the outpatient clinic during pregnancy were approached in the public hospital. This screening strategy was adopted because many un-booked women in public hospitals sought hospital care during delivery, often with a postpartum stay as short as six hours. Furthermore, many of these women did not have documented GDM status. Hence, we decided to recruit only those women from a public hospital who received antenatal care and had complete information. Study details were provided to the potential participants, and the contact information of women who consented to participate was recorded.

In the second stage of screening, women who consented to participate during the first stage were visited at their homes 6 weeks postpartum. During these visits, their oral glucose tolerance test (OGTT) was carried out, and those with fasting blood glucose levels < 120 mg/dl were invited to participate in the study. Those who agreed to participate, written informed consent was obtained, and baseline assessments were completed before the randomization.

### Baseline assessment

At baseline, sociodemographic and obstetric history was collected through face-to-face interviews using a structured questionnaire. In addition, participants were also assessed for their weight, waist and hip circumference, body composition, blood pressure, dietary intake, fasting blood glucose levels, HbA1C, and lipid profile (Additional file 1). Data collectors received training to undertake the measurements.

### Randomization and blinding

After the baseline assessments had been completed, women were randomized to one of the two arms, i.e., intervention or control arm (ratio 1:1). The randomization list was computer generated by a statistician using simple randomization separately for each hospital (public and private). The randomization sequence was kept in sealed opaque envelopes. The allocation of participants to their respective groups (intervention or control) was revealed via telephone to the Community Health Workers (CHWs) who delivered the intervention after the baseline assessments. Independent data collectors who were blind to group allocation collected data at participants’ homes at 6 and 12 months.

### Training of community health workers (CHWs)

Two CHWs received training from a qualified dietitian before delivering the intervention. Three days of face-to-face training (3 h each day) have been conducted at the study hospital. The training sessions mainly focused on lifestyle modification, including a healthy diet, physical activity, and weight reduction and maintenance. On the first day, CHWs received instruction on guiding participants in making healthier food choices, high in fruits, vegetables, and low-fat dietary products, as outlined in the National Health Service (NHS) guidelines [13]. They were also trained to educate participants on portion size control and effective cooking methods. During the second day, CHWs received training on motivating participants to incorporate regular physical activity into their daily routines, emphasizing home-based activities like walking and household chores. In addition, CHWs were encouraged to advise participants about safety measures during the exercise. The other factors covered during the third day included goal setting, action plans, behavior-changing strategies, addressing negative thoughts, and pedometer calibration.

### The intervention

The intervention was centered on equipping individuals with skills, knowledge, and confidence to eat a healthy diet rich in fruit, vegetables, and low-fat dairy products and participate in regular physical exercise to reduce weight (up to 5% of their initial body weight) [12].

The intervention consisted of “face-to-face” consultations at the participant’s home by trained CHWs, as many individuals need a supportive environment to help them get started and make the required changes, mainly if they have been inactive or had previous negative experiences [14]. Participants received an initial consultation of 1.5 h followed by a second consultation of 1 h after 4 weeks (1 month) and 30 min of reinforcement sessions at 3, 6, and 9 months.

### Intervention components

#### Diet and physical activity

During the first consultation, women were provided dietary advice, in line with current guidelines of the National Health Service (NHS) [13], to eat a balanced diet and maintain good health. The focus was on educating women to eat a variety of food in the right amount to achieve a healthy body weight. In addition, they were encouraged to eat high-fiber food, such as fruits, vegetables, whole grains, and lentils, and reduce the amount of refined carbohydrates and saturated fat, including bakery products, deep-fried food, soft drinks, beverages, sweets, and sugars. During the first consultations, women were also encouraged to increase the uptake of physical activity and reduce sedentary behaviors. The consultation thoroughly evaluated the pros and cons of increasing physical activity, addressing obstacles hindering exercise participation. The focus was on enhancing their motivation and self-efficacy and overcoming barriers to adopting a healthy lifestyle.

At months 1, 3, 6, and 9, the consultations provided by the CHWs focused on preventing relapse and encouraging women to continue following healthy eating habits and avoid sedentary behaviors. We extensively addressed effective relapse prevention strategies by helping participants identify potential barriers that might impact their behavior change efforts and helping them overcome these challenges.

#### Pedometers

Participants were provided with a pedometer to serve as a motivational tool that can assist them in quantifying the amount of physical activity they achieved each day/week [15]. In addition, exercise in the form of walking and household chores was particularly encouraged using a pedometer, as this has been shown to increase motivation and achieve a target of 10,000 steps/day [16, 17]. The CHWs also provided support in calibrating pedometers.

#### Calendars and pamphlets

**Fig. 1:**
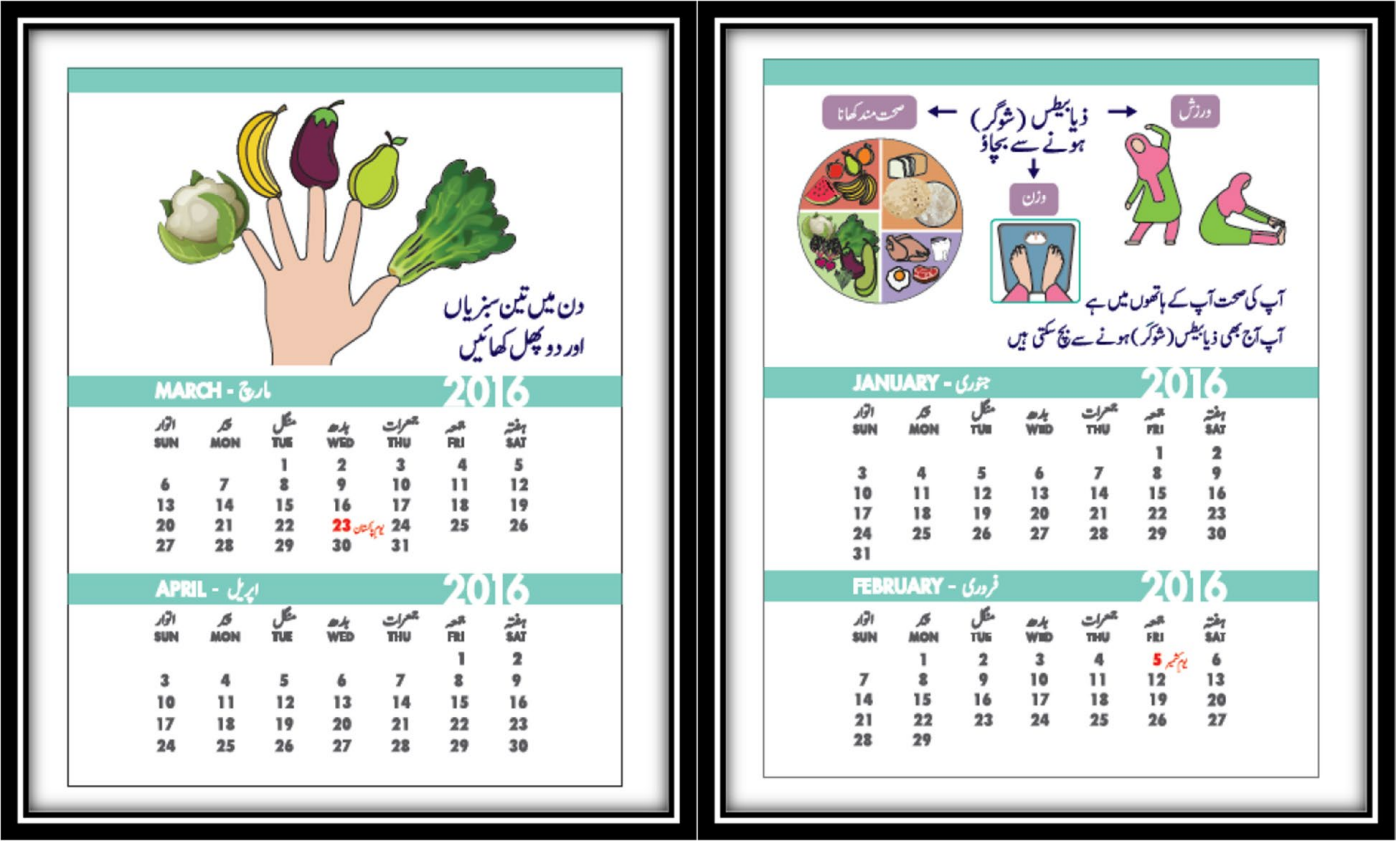
Calendar highlighting healthy diet and physical activities

Participants were provided with calendars and pamphlets during the first consultation. We used visual demonstrations and short messages related to a healthy diet and physical activity due to the participant’s literacy levels. There were six pages in the calendar (2 months per page), with each page containing one message (a total of six messages), starting from prevention of T2DM through lifestyle modifications, then increasing fruits and vegetables in daily diet, avoiding sugary drinks, making 10,000 steps/day a habit, increasing physical activity through household chores, and weight maintenance (Fig. 1).

**Fig. 2:**
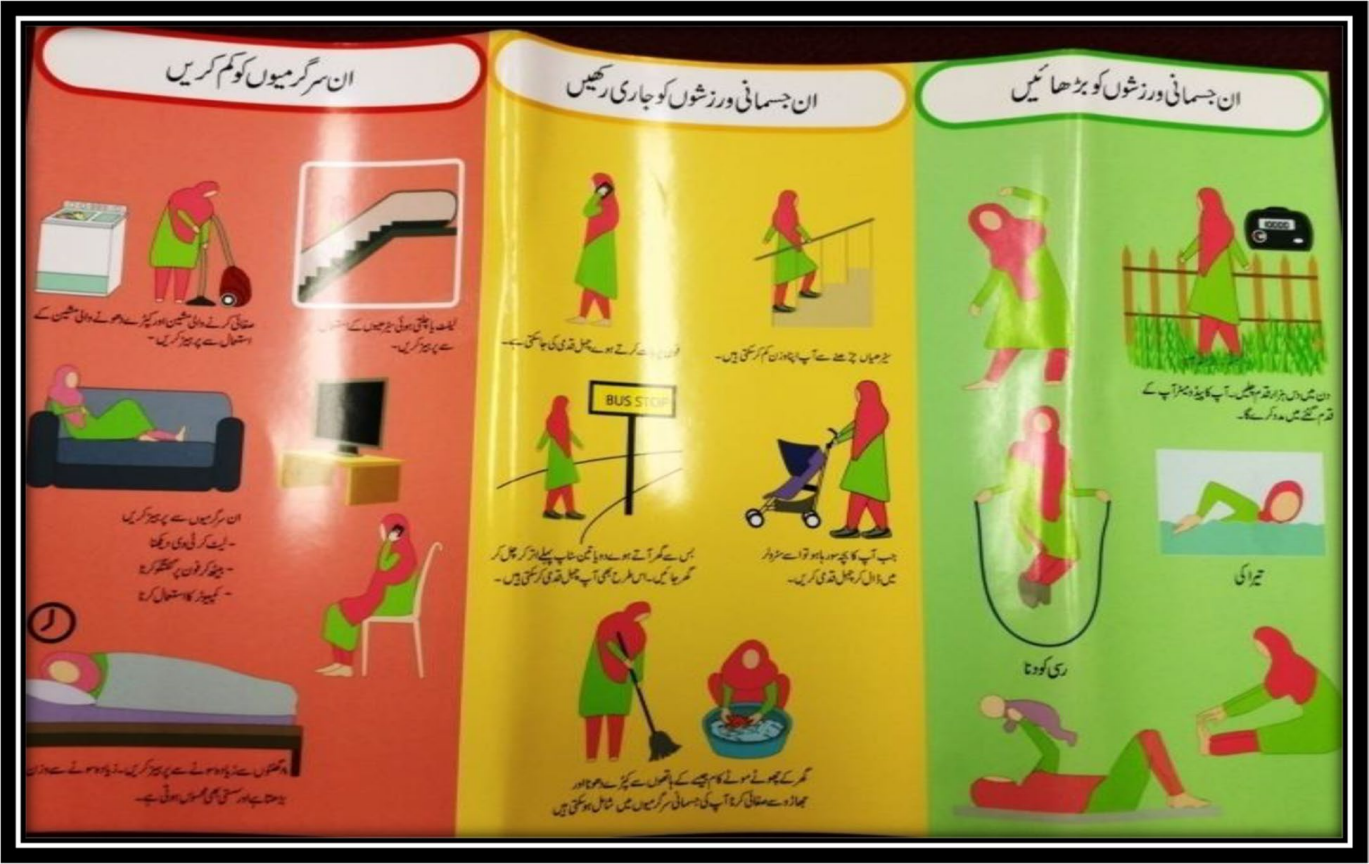
Pamphlets showing a range of home-based physical activities

In addition, due to cultural norms and security concerns, we adopted and promoted home-based physical activity components, such as cleaning the floor and washing clothes by hand, through our pamphlets (Fig. 2). Information about injury prevention, fatigue, and hydration tips during physical activity has also been incorporated.

#### Prerecorded messages and text messages

As m-Health interventions are widely used and have shown some effect on healthcare delivery [18], we sent prerecorded messages and text messages for reinforcement based on themes, such as diabetes, diet, weight, physical activity, and lactation, three times a week. Some examples of prerecorded and text messages are given in Additional file 2.

#### Mobile phones

To minimize attrition, we provided a mobile phone to all women. This approach was needed, as women, particularly from the more deprived parts of Karachi, move and change addresses frequently.

### Control group

Participants followed the usual advice from their care providers.

### Intervention fidelity

Ensuring intervention fidelity involves providing comprehensive training to CHWs before delivering the intervention as well as a refresher training session at 6 months. A training manual was also provided to the CHWs to help them adhere to the standardized protocols throughout the intervention delivery process.

In addition, an independent research officer observed the intervention delivery process to ensure that the CHWs effectively delivered all the planned intervention content.

### Follow-up assessments

Participants were followed up at 6 and 12 months to assess their weight, waist and hip circumference, body composition, and blood pressure. Diet was assessed using a food frequency questionnaire (FFQ) [19] at 6 and 12 months, whereas physical activity was assessed at 6 months using the International Physical Activity Questionnaire (IPAQ) [20]. The laboratory tests, such as fasting blood glucose levels, HbA1C, and lipid profile, were assessed at 12 months (Additional file 1).

### Feasibility outcomes

The feasibility outcomes included the rates of women approached at the two stages of screening, the recruitment rate, and the retention rates of women in the two groups. We explored women’s experiences with the intervention through in-depth interviews. We also compared changes in the anthropometric (weight and body fat %), behavioral (diet), and metabolic outcomes (blood pressure, blood glucose levels, and cholesterol levels) in the two groups at 12 months compared to the baseline that would help us estimate sample size for a future definitive trial.

### Sample size

The study aimed to assess the feasibility of the intervention; therefore, we did not perform a formal sample size calculation; instead, based on Julious and Whitehead’s recommendations, we proposed to recruit at least 160 participants (80 in each arm) to generate variability estimates for our outcomes that would help calculate sample size for a definitive RCT [21, 22]. Since this study involved two-stage screening processes, it was estimated that it would be achieved by approaching 325 women at an initial stage. This estimation accounted for a 50% recruitment rate considering potential refusals, loss to follow-up, and exclusions. However, out of 324 women approached during the screening processes, we were able to recruit 180 participants (88 women in the intervention and 92 in the control arms).

### Study progression criteria for the full trial

We developed the study progression criteria for designing a definitive RCT. This could be determined by achieving at least 70% of the screening rate at the second stage of screening, followed by 50% of the recruitment rate, and an overall loss to follow-up rates between 18 and 29% based on the available literature [23, 24].

### Data analysis

The baseline characteristics of study participants were presented as mean with standard deviation (SD), median with interquartile range (IQR), and frequencies with percentages as appropriate.

The rates of screening, recruitment, and retention were calculated as feasibility outcomes. Dietary data were divided into six food groups (bread/pasta/grains, vegetables, fruits, dairy products, meat/fish/poultry, and desserts), and daily intake was estimated and presented as median and IQR. Three categories of physical activity were developed: inactive, minimally active, and health-enhancing physical activity (HEPA), following the International Physical Activity Questionnaire (IPAQ) data analysis guidelines [25] (Additional file 3).

Changes in the anthropometric (weight and body fat %), behavioral (diet), and metabolic outcomes (blood pressure, blood glucose levels, and cholesterol levels) were presented as mean (SD) at baseline and 12 months in the two groups. Data were analyzed using STATA (V.14.2 StataCorp, TX, USA).

We used a concurrent triangulation design involving quantitative data from the feasibility trial and qualitative data from the interviews. First, each dataset was analyzed separately, and then the findings from the two data sources were triangulated using the study objectives.

### Process evaluation

#### Qualitative interviews

A nested qualitative interview study was conducted at 6 months to explore women’s experiences with the intervention. Out of 81 women from the intervention arm who completed their 6-month follow-up, we randomly approached 10 women for the qualitative interviews. Of these, six women (three from each setting) were agreed and interviewed using in-depth interviews (IDIs). An experienced qualitative researcher conducted the interviews using a semi-structured topic guide, which allowed flexibility in following up on potential areas raised by participants. The interview guide included questions about women’s experience in terms of intervention components, such as home visits, counseling on diet and physical activity, printed material, prerecorded and text messages, mobile phones, and pedometers. The findings of the qualitative study would help refine the intervention and study processes for a future larger trial.

The qualitative data were analyzed based on manual thematic analysis using Excel [26]. All prerecorded interviews were transcribed verbatim into Roman Urdu by the investigators, and all identifiable information was removed. Only the quotes used in this manuscript were translated into English. Two study members examined and identified the codes and organized them according to the themes, i.e., strengths, weaknesses, and suggestions for improving the intervention. After the thorough review of the theme content by the study team, areas of consensus and differences in the opinion of the participants were finalized and defined with the corresponding quotes.

## Results

### Primary findings

#### Feasibility of participant screening, recruitment, and retention rates

**Fig. 3:**
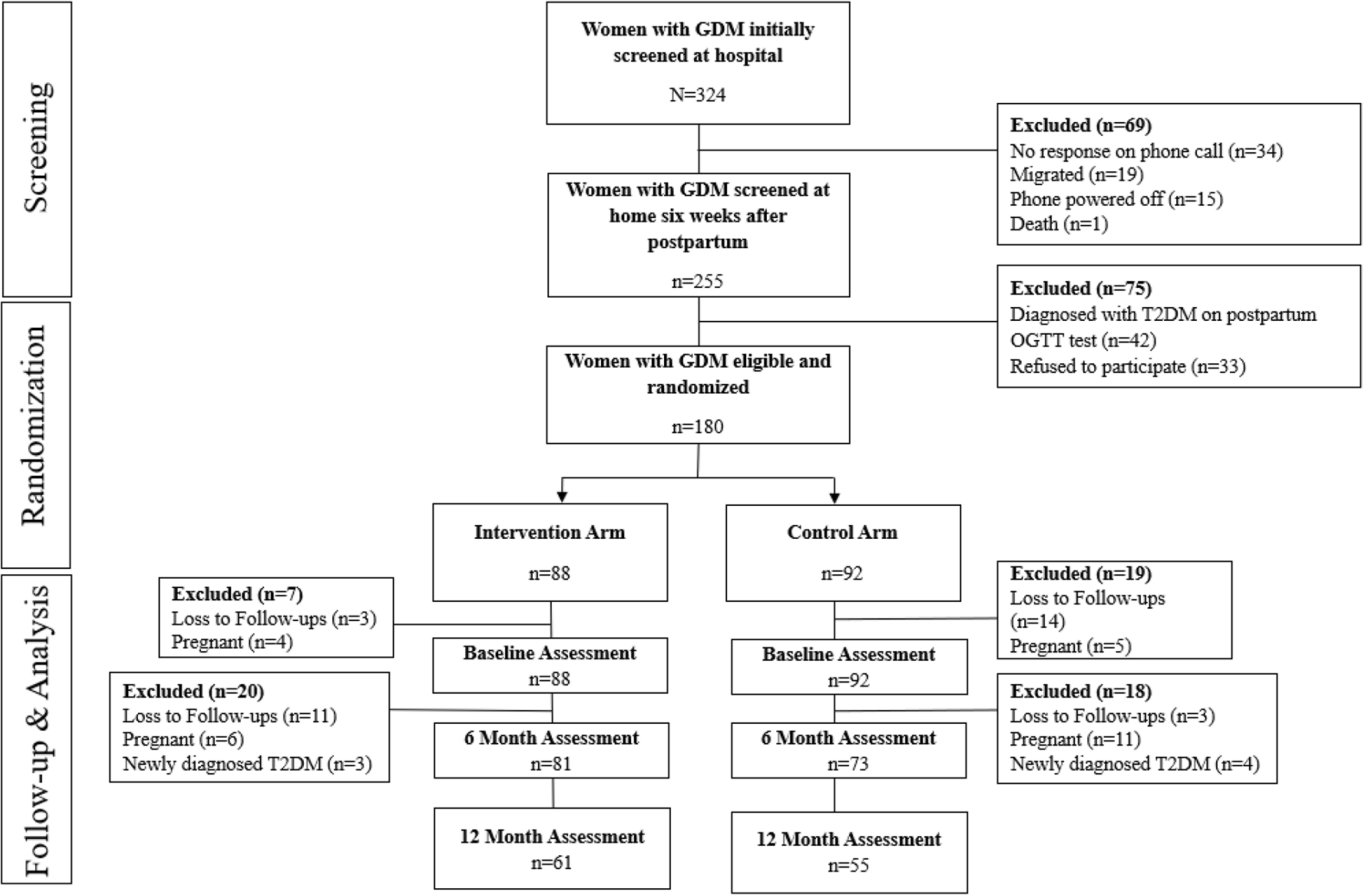
Participant flow diagram. T2DM = type 2 diabetes mellitus, OGTT = oral glucose tolerance test

Figure 3 describes participant flow throughout the study and the reasons for their exclusions and drop-outs. During the first stage of screening, 324 women were identified with GDM from the two hospitals (one public and one private). Out of the 324 potential GDM women identified and consented initially, we were able to contact 255 women (78.7%) 6 weeks postpartum. The reasons for not establishing contact included unresponsive phone calls (*n* = 34; 49%), migration (*n* = 19; 27.5%), phone powered off (*n* = 15; 21.7%), and death (*n* = 1; 1.5%). Of the 255 women assessed for their eligibility at home, 180 (70.6%) women were eligible and randomized to one of the two arms, i.e., intervention (*n* = 88) and control (*n* = 92). Forty-two women (56%) were diagnosed with T2DM on the oral glucose tolerance test (OGTT), while 33 (44%) refused to participate during the second step of screening.

During the study period, we lost 20 (22.7%) participants in the intervention arm and 17 (18.5%) in the control arm. We excluded 13 (14.8%) participants due to subsequent pregnancies (*n* = 10, 11.4%) and newly diagnosed diabetes (*n* = 3, 3.4%) in the intervention arm. The exclusion in the control arm was 20 (21.7%) due to subsequent pregnancies (*n* = 16, 17.4%) and newly diagnosed diabetes (*n* = 4, 4.3%).

#### Baseline characteristics

Both groups were comparable in age, sociodemographic characteristics, household size, and religion. Approximately 69% of the women in the intervention arm were multiparous compared to 58% in the control arm, whereas grand multiparas were more common in the control arm (intervention 10.2%, control 16.3%). History of pregnancy-induced hypertension was present in 21 (23.9%) women in the intervention arm and 33 (35.9%) in the control arm (Table 1).

**Table 1:** Baseline characteristics of the study participants

Variables	Intervention arm (*n* = 88) *n* (%) or mean ± SD or median (IQR)	Control arm (*n* = 92) *n* (%) or mean ± SD or median (IQR)
Age (years)	30.2 ± 4.8	30.7 ± 5.5
Number of years of schooling	12.1 ± 4.4	11.5 ± 5.1
Number of years of schooling of husband	12.5 ± 4.4	12.0 ± 4.7
Number of household members	7.1 ± 3.8	6.6 ± 3.3
Number of earning members in the household	2.0 ± 1.4	2.0 ± 1.5
Spending on food (USD)	175 (100, 250)	150 (100, 287.5)
Spending on health (USD)	4 (0, 30)	0 (0, 30)
Spending on education (USD)	20 (0, 80)	15 (0, 80)
Religion		
Muslims	86 (97.7)	87 (97.7)
Others	2 (2.3)	2 (2.3)
Primipara	18 (20.5)	23 (25.0)
Multipara	61 (69.3)	54 (58.7)
Grand multipara	9 (10.2)	15 (16.3)
History of miscarriage	40 (45.5)	46 (50.0)
History of hypertension	7 (8.0)	13 (7.2)
History of pregnancy-induced hypertension	21 (23.9)	33 (35.9)
Number of live births	2.3 ± 1.4	2.4 ± 1.9

### Secondary findings

#### Anthropometric, behavioral, and metabolic outcomes

Means and SDs for all study outcomes at baseline and 12 months between the two groups are presented in Table 2. At the end of 12 months, the weight reduction in the intervention arm was 0.6 kg (68.5 vs. 67.9). In contrast, weight in the control arm increased by 0.4 kg (68.5 vs. 68.9). Similarly, systolic and diastolic blood pressure in the intervention arm was reduced by 1.4 and 0.7 mmHg, respectively; however, there was no change in the blood pressure in the control arm. While comparing laboratory parameters, the intervention and control arms did not differ at baseline and end line for most parameters, such as fasting blood glucose levels and HbA1c. However, the cholesterol levels varied between the two groups, with a greater reduction in the intervention arm for serum cholesterol (− 2.7 mg/dl), serum triglycerides (− 5.5 mg/dl), and serum LDL levels (1.2 mg/dl). At the end of 12 months, 8% (*n* = 7) of women in the intervention arm and 14% (*n* = 13) in the control arm developed T2DM.

**Table 2:** Change in outcomes after 12 months

Variables	Baseline		12 months	
	Intervention *n* = 88 Mean ± SD	Control *n* = 92 Mean ± SD	Intervention *n* = 61 Mean ± SD	Control *n* = 55 Mean ± SD
Anthropometric outcomes				
Weight (kg)	68.5 ± 13.5	68.5 ± 15.3	67.9 ± 15.2	68.9 ± 14.9
BMI	27.5 ± 5.1	27.8 ± 5.5	27.2 ± 6.0	28.1 ± 5.6
WHR	0.82 ± 0.06	0.82 ± 0.07	0.84 ± 0.07	0.84 ± 0.06
Body fat %	35.1 ± 7.5	35.4 ± 8.0	33.8 ± 9.8	35.0 ± 8.2
Visceral fat (%)	5.2 ± 2.7	5.6 ± 3.1	5.6 ± 3.3	5.5 ± 3.1
Metabolic outcomes				
Systolic BP	107.8 ± 13.9	108.7 ± 13.2	106.4 ± 12.4	108.8 ± 11.9
Diastolic BP	75.8 ± 9.4	77.7 ± 8.7	75.1 ± 9.0	77.8 ± 9.6
Fasting plasma glucose (mg/dL)	86.7 ± 9.5	86.7 ± 10.2	96.0 ± 27.8	95.8 ± 23.3
HbA1C (%)	5.8 ± 0.47	5.8 ± 0.39	6.1 ± 1.1	6.1 ± 0.8
Serum cholesterol	177.1 ± 28.9	181.7 ± 41.7	164.4 ± 23.9	171.7 ± 37.3
Serum triglycerides	143.3 ± 99.7	133.5 ± 77.8	123.7 ± 91.7	119.4 ± 72.3
Serum HDL	47.1 ± 13.6	46.6 ± 10.5	45.4 ± 10.7	46.2 ± 10.6
Serum LDL	112.8 ± 29.0	118.8 ± 30.9	100.6 ± 23.1	107.8 ± 30.9
Behavioral outcome (dietary intake^a^)				
Bread/pasta/grains	3.5 (2.8, 4.4)	3.9 (2.8, 5.1)	3.0 (2.0, 4.0)	3.4 (2.1, 5.4)
Vegetables	0.92 (0.65, 1.3)	0.88 (0.61, 1.4)	0.77 (0.62, 1.1)	0.72 (0.62, 1.1)
Fruits	0.59 (0.31, 1.2)	0.70 (0.07, 2.0)	0.52 (0.30, 0.68)	0.65 (0.39, 1.4)
Dairy products	1.0 (0.32, 1.6)	1.0 (0.23, 1.4)	0.60 (0.23, 1.1)	0.44 (0.12, 1.1)
Meat/fish/poultry	0.92 (0.67, 1.6)	1.2 (0.78, 1.8)	0.78 (0.55, 1.2)	0.78 (0.46, 1.5)
Desserts	0.24 (0.14, 0.36)	0.19 (0.12, 0.36)	0.17 (0.09, 0.27)	0.21 (0.14, 0.34)

*BMI* body mass index, *WHR* waist-to-hip ratio, *BP* blood pressure

^a^Median (IQR)

For diet, no differences in the reduction of dietary intake were seen among the two groups at the end of 12 months except for desserts, which were higher in the control arm (Table 2). Physical activity was assessed at 6 months, revealing that in the intervention arm, fewer women were inactive (*n* = 24, 30%) compared to the control arm, where nearly half of the women were inactive (*n* = 35, 48.6%). In addition, two participants from the intervention arm reported vigorous physical activity, whereas none of the participants from the control group were involved in vigorous physical activity (Additional file 4).

## Process evaluation

The findings from the qualitative study are as follows:

### Strengths of the intervention

Women appreciated the provided calendar as a good information resource and reminder for their exercise and healthy diet.

“Calendar was in front of us and served as a reminder.”

(Participant 012; age: 29 years old; public hospital)

“The calendar you provided has reminders set on it. The pages that have been turned over also become a flashback.”

(Participant 064; age: 35 years old; private hospital)

Home visits and blood tests were also welcomed, as women consider it a general health check-up at their doorsteps.

“Routine check-up was done, and GDM field visitors provided services that provided knowledge also.”

(Participant 004; age: 32 years old; private hospital)

“It was a facilitator for me and saved the cost of the test (laboratory investigation).”

(Participant 044; age: 26 years old; public hospital)

The experiences regarding prerecorded messages and mobile phones differed based on socioeconomic background. For example, women recruited from a public hospital with a population of lower socioeconomic status valued extra cell phones and prerecorded messages. One woman said:

“Cell phone was a benefit because we were in contact for diet and exercise-related things.”

(Participant 013; age: 30 years old; public hospital)

### Weaknesses of the intervention

Women’s experiences were unsatisfactory with pedometers, and they considered them a burden rather than a facilitator.

“There were no special feelings for it. It was like if I am going to wear it, then I have to walk, and it will record also.”

(Participant 046; age: 30 years; private hospital)

"It was misused as I used it less, and children played with it like a toy."

(Participant 012; age: 29 years; public hospital)

Women expressed dissatisfaction with the pamphlets for dietary advice and physical activity as they misplaced the pages. In addition, they did not benefit from study phones, expressing difficulties managing two phones (personal and study phone). Additionally, they highlighted concerns about the potential misuse of the study phones by family members at home.

‘There was no benefit of the phone; it was there but was not in my use.”

(Participant 064; age: 35 years old; private hospital)

“Children used to play games sometimes [on cell phones].”

(Participant 012; age: 29 years old; public hospital)

The timing of the prerecorded messages was also not suitable for women, as it was late afternoon (between 2 and 3 pm), which was resting time for them and their babies. In addition, women were not satisfied with the timing of the home visits, as they were conducted early in the morning.

“As soon as I put my baby to sleep, approximately 2 pm or 4 pm, the phone used to start ringing repeatedly.”

(Participant 046; age: 30 years old; private hospital)

“Your team used to visit early in the morning and give lectures, so I had to get up early to clean the house, which greatly irritated me.”

(Participant 013; age: 30 years; public hospital)

### Suggestions for the improvement of the intervention

Calendars came out as a strength of the intervention. However, participants were advised to replace wall calendars with table calendars and make them more visual due to a concern that hanging them could reveal their personal information to anyone coming home. They also recommended incorporating all necessary messages in the calendar rather than providing it separately as printed material (pamphlets).

“I did not put it (Calendar) on the wall because whoever walks in would have known I have GDM. Table calendars should be provided so they can be placed on the side near the bed.”

(Participant 004; age: 32 years; private hospital)

‘Increase pictures of messages other than an exercise in the calendar, like the use of vegetables as per budget.”

(Participant 013; age: 30 years old; public hospital).

Women proposed increasing the frequency of home visits from once monthly to bimonthly; however, they recommended visiting homes during the afternoon hours.

‘Home visits should be increased to twice a month.”

(Participant 044; age: 26 years old; public hospital)

Women preferred text messages and suggested sending these messages in the morning hours with the frequency of once weekly.

“It should be only once a week. Because listening to the same message again and again is irritating.”

(Participant 004; age: 32 years old; private hospital)

## Discussion

The postpartum period is considered a “window of opportunity” to intervene in women with a history of GDM to prevent T2DM [22]. This 1-year feasibility study targeted postpartum women with a history of GDM to prevent T2DM to inform the development of a contextually relevant definitive RCT in LMICs. The proposed lifestyle modification intervention was feasible for women with GDM who continued the study. During the IDIs, we found continuous counseling on diet and physical activity, home visits, and calendars to be the strengths of the intervention. However, we faced challenges in contacting women postpartum due to the time lag between the first contact at the hospital during the initial screening and the second contact at home 6 weeks after delivery, with a screening rate of 78.7% at the second stage of screening. The recruitment rate in our study was 70.6%, with an overall attrition rate of 20.6%. The findings of the feasibility study supported the design of a definitive RCT.

We provided a comprehensive lifestyle modification intervention involving home-based, face-to-face counseling, print material, prerecorded messages, text messages, pedometers, and cell phones to promote lifestyle modification among women with a history of GDM to prevent T2DM. Women expressed satisfaction with the efforts made by our study team in providing home-based follow-up visits and counseling sessions. The convenience and individualized nature of the intervention were well received by the women, and they recommended increasing the frequency of home visits from once to twice a month. However, they requested that the timing of home visits be changed from morning to afternoon.

We found that short messages with pictorial demonstrations incorporated into the calendar were the strength of the intervention as it worked as a reminder. However, women recommended replacing the wall calendar with a table calendar to maintain the confidentiality of their GDM diagnosis with others. In addition, women suggested incorporating all necessary messages into the calendar rather than providing them separately as pamphlets.

Moreover, women expressed dissatisfaction with some components of the intervention, such as prerecorded messages, pedometers, and study cell phones, as they found it to be burdensome rather than supportive. They preferred text messages instead of prerecorded messages with a frequency of once weekly. Similarly, Tewari et al. supported face-to-face counseling sessions augmented with pictorial material, text messages over prerecorded voice messages, and social support to reinforce adopting a healthy lifestyle during the postpartum period [27].

Similar to other studies, we encountered challenges related to recruiting and retaining women with recent GDM [23, 24]. For example, at the first screening stage, we screened women for their GDM status at public and private hospitals during antenatal and intrapartum visits, respectively, and sought their consent. In the second stage, women who consented were approached 6 weeks postpartum at their homes to assess eligibility for enrollment. Due to the time lag between the two screenings, we could not reach out to all GDM women who initially agreed and consented due to unresponsive phone calls, migration, and phone switched-off issues.

The loss to follow-up in our study (20.5%; *n* = 37) was comparable with other similar studies that reported loss to follow-up rates ranging from 18 to 29% [23, 24]. We could not identify reasons for our loss to follow-up; however, we believe it to be random, as it occurred at a similar rate in both arms. Other studies from Australia reported that family responsibilities and women returning to work were the most common reasons for loss of follow-up [23, 24]. In addition, a higher number of women in our study were excluded (*n* = 33) due to subsequent pregnancies (*n* = 26) and the incidence of T2DM (*n* = 7) during the study follow-ups. O'Reilly SL et al. also found a higher proportion of women excluded from the study due to subsequent pregnancies [28]. Moreover, we encountered difficulties recruiting participants from the public hospital, as it serves a deprived catchment area. It has also been observed that individuals from lower socioeconomic strata in Pakistan frequently change residences and have multiple contact numbers. Therefore, despite our utmost efforts, we faced challenges following up with the women initially recruited.

A meta-analysis presented behavioral, anthropometric, and metabolic outcomes among women with prior GDM who received lifestyle modification intervention [29]. Favorable evidence regarding changes in dietary patterns, limited evidence for changes in anthropometric and physical activity outcomes, and no evidence for changes in glycemic indicators have been reported in the meta-analysis [29]. Our study was designed primarily to inform the structure of a definitive trial rather than being statistically powered to demonstrate conclusive evidence of the intervention's effectiveness. However, we estimated the change in anthropometrics and laboratory parameters at 12 months to estimate a sample size for a future definitive trial. Our study findings demonstrated a considerable reduction in anthropometry (weight) and metabolic indicators (blood pressure and serum cholesterol levels) among women in the intervention arm compared to the control arm. Unfortunately, no differences in the changes in glycemic parameters (fasting blood glucose and HbA1c) or diet were observed between the two groups. In addition, we found that more women in the control arm were inactive at 6 months than women in the intervention arm.

Our study has several strengths. To the best of our knowledge, this is one of the few studies in Pakistan targeting women with a history of GDM during the postpartum period to prevent T2DM through lifestyle modification and behavior change. Despite several challenges in recruitment and follow-up, women from both public and private hospitals were recruited. Furthermore, a home-based comprehensive lifestyle modification program was delivered to women through CHWs, making it convenient and easily accessible. We highlighted contextually relevant physical activity through household chores, making it adaptable and sustainable. To mitigate interviewer bias, independent data collectors collected data during the follow-up visits (6 and 12 months).

There are some limitations; despite adopting strategies, for example, providing cell phones to enrolled women, the attrition rate was high. This feasibility study was designed to inform the planning of a definitive trial, and hence, sample size and statistical power were not primary concerns. However, it is crucial to consider a higher attrition rate while estimating the sample size for a future larger trial to make it sufficiently powered to evaluate the intervention effectively. In addition, it is important to note that the loss to follow-up was similar in the two groups. We measured physical activity only at 6 months and could not compare whether the activity levels were comparable between the two groups at baseline. In addition, we were unable to compare changes in the physical activity levels over a longer period.

## Conclusions

This feasibility shows that delivering a home-based lifestyle modification prevention program to women with a history of GDM is feasible. CHWs play an integral role in supporting the healthcare system of Pakistan, specifically in improving maternal and child health. Increasing their capacity and capability would help strengthen healthcare delivery within communities and the primary prevention of T2DM.

### Supplementary Information

Additional file 1. Measurements and tools.

Additional file 2. Prerecorded messages and text messages.

Additional file 3. Physical activity categories using IPAQ.

Additional file 4. Physical activity levels at 6 months.

## Data Availability

The datasets used and/or analyzed during the current study are available from the corresponding author upon reasonable request.

## Abbreviations

GDM: Gestational diabetes mellitus
T2DM: Type 2 diabetes mellitus
LMICs: Low- and middle-income countries
RCT: Randomized controlled trial
CHWs: Community health workers
NHS: National Health Service
SD: Standard deviation
IQR: Interquartile range
HEPA: Health Enhancing Physical Activity
IPAQ: International Physical Activity Questionnaire
IDIs: In-depth interviews
OGTT: Oral glucose tolerance test
BMI: Body mass index
WHR: Waist-to-hip ratio
BP: Blood pressure
