# Supplementary material for: Conducting a diabetes mellitus prevention trial in women with GDM in Pakistan: a feasibility study
Source: Pilot Feasibility Stud. 2024 Jun 15;10:92. doi: 10.1186/s40814-024-01514-3 (PMC11179295; doi:10.1186/s40814-024-01514-3)
Supplement: Supplementary file 1 — Additional file 1. Measurements and tools. [file 40814_2024_1514_MOESM1_ESM.docx]

**Additional file 1: Measurements and tools:**

| **Measurements** | **Tools** | **Assessments time points** | | |
| --- | --- | --- | --- | --- |
|  |  | **Baseline** | **Follow-ups** | |
|  |  | **Month 0** | **Month**  **6** | **Month**  **12** |
| **Anthropometry** | | | | |
| Height |  | X |  |  |
| Weight |  | X | X | X |
| Waist circumference |  | X | X | X |
| Hip circumference |  | X | X | X |
| **Body composition** | | | | |
| Body fat | Tanita Body Composition Analyzer- (SC 330ST) | X | X | X |
| Body water |  | X | X | X |
| Visceral fat |  | X | X | X |
| Muscle mass |  | X | X | X |
| Bone mass |  | X | X | X |
| **SBP and DBP** |  | X | X | X |
| **Laboratory investigations** | | | | |
| Fasting plasma glucose | Aga Khan Laboratories (accredited by the College of American Pathologists) | X |  | X |
| HbA1C |  | X |  | X |
| Serum cholesterol |  | X |  | X |
| Serum triglycerides |  | X |  | X |
| Serum LDL |  | X |  | X |
| Serum VLDL |  | X |  | X |
| **Diet intake** | Semi-structured Food Frequency Questionnaire (FFQ) | X | X | X |
| **Physical activity level** | International Physical Activity Questionnaire (IPAQ)-short |  | X |  |

**SBP**= Systolic Blood Pressure, **DBP**= Diastolic Blood Pressure
