# Supplementary material for: Conducting a diabetes mellitus prevention trial in women with GDM in Pakistan: a feasibility study
Source: Pilot Feasibility Stud. 2024 Jun 15;10:92. doi: 10.1186/s40814-024-01514-3 (PMC11179295; doi:10.1186/s40814-024-01514-3)
Supplement: Supplementary file 2 — Additional file 2. Prerecorded messages and text messages. [file 40814_2024_1514_MOESM2_ESM.docx]

**Additional file 2: Prerecorded messages and Text messages**

| **Theme 1: Diabetes** | |
| --- | --- |
| **Message 1:**  Asalaam o Alaikum. Ye message apko Aga Khan University Hospital aur Jinnah hospital ki taraf se bheja ja raha hai kya ap jaante hain k Jin khawateen ko hamal kay doraan diabetes hoti hai, un main type 2 diabetes K imkanaat satar feesat (70%) barh Jaatay hain. Behtar aur sehat mand ghiza, Jismaani warzish our wazan per kaaboo rakh ker ap type 2 diabeter (sugar) k khatray se bach sakte hain. Mazeed maloomat k leye apni Aga khan university or Jinnah hospital ki taraf se di hui sim se 331043 se 9 se 5 ky darmeyan milaayen or humary tool free call per maaloonlmat Haasil keren. Ya phur kisi number se 0302-8722923 millaayen. | **Message 2:**  Asalaam-o-Alaikum. Ye message ap ko Aga Khan University Hospital aur Jinnah hospital ki taraf se bheja Ja raha hai. Diabetes se muraad khoon mein glucose ya sugar ka barh Jana hai. Bhook aur pyaas ka ziada lugna, baar baar aur khasoosan raat mein pishaab ana, thakan ziada mehsoos hona, or wazan ek dam barhna ya kam hona diabetes ki alamaat hain. Agar ap ko koi bhi alamaat hon tu foran doctor se rujoo karen, Mazeed maloomat k leye subah 9 se 5 bajay k darmeyaan apni di hui sim se 331043 milaayen aur muft call per apnay sawaalon k Jawaab paayen. |
| **Theme 2: Diet** | |
| **Message 1:**  Asalaam o Alaikum. Ye call ap ко Aga Khan hospital or Jinnah hospital ki taraf se bheji ja rahi hai. Kya ap jaanti hain k ap ki ghiza sehat per asar andaaz hoti hai? Is baat ka khaas Khayaal rakhen kay ek waqt kay khaanay mein plate ka aadha hisa phal (fruits) ya sabzi ho. Ye phal ya sabzi mausam ky hisaab se koi bhi ho sakti hai ju ap kay jaib kharch kay mutabiq ho. Apni ghizaa mein phal or sabzi ka shumaar Ker k ap taaza dam or sehat mand mehsoos karen ge. Ghiza k baaray mein mazed maloomaat k leye subah 9 se 5 bajay k darmeyaan hamary toll free number 331043 per call karen. | **Message 2:**  Kya apki plate ka ziada hissa roti ya chawal per mushtamil tou nahin hai? kya ap ko maaloom hai ziada miqdaar mein roti ya chawal motaapay ka sabab ban sakte hain? Isi leye is baat ka khayal rakhen ky roti ya chawal ka hissa plate kay ek tehai (1/3) hissay se ziada na ho. Mazeed malomaat k leye Subah 9 se 5 bajay k damayaan humaray toll free no 331043 per call karen aur apnay sawaaloon ke jawab paayen. |
| **Theme 3: Weight** | |
| **Message 1:**  Asalaam o Alaikum. ye message ap ko Aga Khan Hospital aur Jinnah hospital ki taraf se bheja ja raja hai. Kya ap apnay wazan per qari nazar rakhti hain? Haftay mein ek baar apna wazan zaroor karen. Jab ap dekhen k ap k wazan mein tabdeeli aa rahi hai tou ap warzish aur chehal qadmi mai aur dilchaspi len gi. Is baaray mein Mazeed maaloomaat k leye subah 9 se 5 bajay k darmayaan apni di hui sim se humaray toll free number 331043 pe call karen. | **Message 2:**  Asalaam o Alaikum. ye message ap ko Aga Khan Hospital aur Jinnah hospital ki taraf se bheja ja raja hai. Kya ap ko maloom hai k agar ap k pait k hissay mein charbi jama ho gi tu ye diabetes honay kay imkaanaat barha sakti hai? Is baat ka khaas khayaal rakhen k ap kamar ki golai naapen aur jism k is hissay mein charbi na jama honay den. Is baaray mein Mazeed maaloomaat k liay subah 9 se 5 bajay k darmayaan apni di hui sim se humaray toll free number 331043 pe call karen. |
| **Theme 4: Physical Activity** | |
| **Message 1:**  Asalaam o Alaikum. ye message ap ko Aga Khan Hospital aur Jinnah hospital ki taraf se bheja ja raja hai. Sehat mand aur tandrust rehnay k leye zaroori hai k ap wazan per kaaboo rakhen, jis ka behtareen tareeqaa warzish aur chehal qadmi hai. Hamara mashwara ye hai k ap roz kamazkam 30 minute tak jismaani warzish keren, ya 10,000 qadam chalen. Ap ka pedometer ya step counter ap ko agaah rakhay ga k aj ap kitnay qadam chali hain. Mazeed maaloomaat k leye subah 9 se 5 bajay k darmayaan apni di hui sim se humaray toll free number 331043 pe call karen. | **Message 2**:  Asalaam o Alaikum. ye message ap ko Aga Khan Hospital aur Jinnah hospital ki taraf se bheja ja raja hai. Kya ap ko 30 minute paidal chalna dushwaar lagta hai? Humaray paas is ka hal mojood hai. Ap din mein 3 martaba 10 minute k liay paidal chalen. Ye mushkil bhi nahin ho ga aur is tarha ap ko chehal qadmi ki aadat bhi ho jayay ge. Warzish ke baray main mazeed maaloomaat ky leye subah 9 se 5 bajay k darmayaam apni di hui sim se humaray toll free number 331043 pe call karen. |
| **Theme 5: lactation** | |
| **Message 1:**  Asalaam o Alaikum. ye message ap ko Aga Khan Hospital aur Jinnah hospital ki taraf se bheja ja raja hai. Ap k bachay k leye bohat zaroori hai k ap us ko apna doodh pilaayen. Agar ap ghar se bahar kaam kerti hain tu apna doodh ghar pe kisi bottle mein nikaal k jaayen taakay koi ap k bachay ko wo pilaa sakay. Apna doodh pilaanay se na tou sirf ap ka bacha sehat mand rahe ga, balkay ap ko wazan ghataanay main bhi madad milay ge. Mazeed maaloomaat k leye subah 9 se 5 bajay k darmayaan apni di hui sim se humaray toll free number 331043 pe call karen. | **Message 2:**  Asalaam o Alaikum. ye message ap ko Aga Khan Hospital aur Jinnah hospital ki taraf se bheja ja raha hai. Bachay ki pedaaish k baad jo pehla doodh banta hai wo ap k bachay ki sehat k leye bohat eham hai. Is se bachay k haazmay mein madad milti hai aur us ko mukhtalif beemarriyon aur jaraseem se bachaya ja sakta hai. Agar ap ko lagta hai k doodh ki miqdaar kaafi nahin hai tu yaad rakhen k jitna doodh ap apnay bachay ko us ki bhook k mutaabiq pilaayen ge utna hi qudrati tor per doodh zyada banay ga. Mazeed maaloomaat k leye subah 9 se 5 bajay k darmayaan apni di hui sim se humaray toll free number 331043 pe call karen. |
