# Supplementary material for: Conducting a diabetes mellitus prevention trial in women with GDM in Pakistan: a feasibility study
Source: Pilot Feasibility Stud. 2024 Jun 15;10:92. doi: 10.1186/s40814-024-01514-3 (PMC11179295; doi:10.1186/s40814-024-01514-3)
Supplement: Supplementary file 3 — Additional file 3. Physical activity categories using IPAQ. [file 40814_2024_1514_MOESM3_ESM.docx]

**Additional file 3: Physical activity categories using IPAQ**

| **Categories** | **Description** |
| --- | --- |
| **Inactive** | < 3 days of vigorous activity of at least 20 minutes per day  OR  < 5 days of moderate-intensity activity or walking of at least 30 minutes per day |
| **Minimally Active** | Three or more days of vigorous activity of at least 20 minutes per day  OR  Five or more days of moderate-intensity activity or walking of at least 30 minutes per day |
| **HEPA active** | Approximately at least 1.5 -2 hours of total activity per day, of at least moderate intensity activity |

**HEPA**= Health-enhancing physical activity, **IPAQ**= International Physical Activity Questionnaire
