# Supplementary material for: Conducting a diabetes mellitus prevention trial in women with GDM in Pakistan: a feasibility study
Source: Pilot Feasibility Stud. 2024 Jun 15;10:92. doi: 10.1186/s40814-024-01514-3 (PMC11179295; doi:10.1186/s40814-024-01514-3)
Supplement: Supplementary file 4 — Additional file 4. Physical activity levels at 6 months. [file 40814_2024_1514_MOESM4_ESM.docx]

**Additional file 4: Physical activity levels at 6 months**

| **Physical activity levels** | **Intervention arm (n= 80)**  **n (%)** | **Control arm (n= 72)**  **n (%)** |
| --- | --- | --- |
| HEPA active | 2 (2.5) | 0 |
| Minimally active | 54 (67.5) | 37 (51.4) |
| Inactive | 24 (30) | 35 (48.6) |

**HEPA**= Health-enhancing physical activity
